# Analysis of a Measurement Method and Test System for Pressure Change Rates in Commercial Vehicle Brake Chambers

**DOI:** 10.3390/s22093427

**Published:** 2022-04-30

**Authors:** Gangyan Li, Rui Shen, Yudong Liu, Fan Yang, Jian Hu

**Affiliations:** School of Mechanical and Electronic Engineering, Wuhan University of Technology, 122 Luoshi Road, Wuhan 430070, China; gangyanli@whut.edu.cn (G.L.); 270951@whut.edu.cn (R.S.); 18571492736@163.com (Y.L.); yang_fan@whut.edu.cn (F.Y.)

**Keywords:** commercial vehicle, brake chamber, braking comfort, pressure change rate, test system

## Abstract

The pressure change rate (PCR) of the brake chamber is the key control parameter and evaluation index in the pneumatic braking system for intelligent braking. The PCR threshold value of commercial vehicle brake chambers for braking comfort is analyzed. The PCR measurement method based on a laminar flow resistance tube is proposed, and the PCR test system is designed. The simulation model of a PCR test system for commercial vehicle brake chambers is presented. By analyzing the simulation and experimental results, it is validated that the PCR test system of commercial vehicle brake chambers has the function of measuring PCR in real time. Finally, according to the MSA (Measurement System Analysis) evaluation method, the performance of the PCR test system for commercial vehicle brake chambers is analyzed, and the correctness and applicability of the test system are verified.

## 1. Introduction

Pneumatic braking is widely used in commercial vehicles because of its advantages such as a high braking efficiency, high reliability and continuous braking in the case of power source failure. A vehicle’s intelligent braking requires that the pneumatic braking system of commercial vehicles needs to reach the specified braking pressure within the specified time. The dynamic characteristics of the pneumatic braking system need to consider the braking pressure response and braking time response at the same time, so as to eliminate or reduce the pressure deviation and time deviation. Therefore, the pressure change rate (PCR), considering both the braking pressure response and braking time response, is used as the control parameter of electronically controlled pneumatic braking systems. By controlling the PCR, the braking pressure of the brake chamber can be changed as expected, and the stability, safety and comfort of the commercial vehicle during braking are guaranteed. The PCR of brake chambers is the key control parameter and evaluation index of pneumatic braking systems for intelligent braking. Real-time measurement of PCR for brake chambers is the basis of accurate electronically controlled pneumatic braking systems.

In terms of brake chamber pressure responses, Lu, Y. [1] and Ma, E. [2] concluded through simulation analysis and experimental verification that the main cause of response lag in pneumatic braking systems is the deformation of the rubber diaphragm of the diaphragm brake chamber, and the response lag time of the system is tens of milliseconds. Zhou, J.W. [3] built the Simulink simulation model of the spring brake chamber, set the parameters of each component of the brake chamber, simulated and analyzed the response curve and push rod movement change rule of the spring brake chamber under different working states, and verified the correctness of the simulation model. Ma, Z [4] and Lu, Y. [5] designed algorithms to estimate the pusher travel and found that the pusher travel affects not only the wheel braking force, but also the response time of the brake chamber. The longer the brake chamber pusher travel, the slower the response time. In the simulation model of brake chambers, Palanivelu, S. [6] derived the mathematical models of the series double chamber brake valve, the relay valve and the brake chamber in the pneumatic braking system, and established the models of the braking system components. Based on this model, they studied the effects of different parameters on the performance of the braking system, such as the response time, apply rate release rate, deceleration and stopping distance. Yang, F. et al. [7] solved the partial differential control equation of the tubeline based on the constrained interpolation curve method, and set the boundary condition with the brake chamber model, obtaining the rule of the influence of the tubeline on the brake chamber’s pressure response: the brake pressure response time increased significantly with the pipe length, but the diameter of a pipe has less effect when the diameter is in the range of 7.5–12 mm. The length of the tubeline and the size of the brake chamber inlet are the main reasons for the delay in the brake chamber pressure response. In order to verify the correctness of the brake chamber mathematical model and simulation model, Meng, W.X. [8] established the simulation model of the pneumatic ABS (Antilock Brake System) regulator and the brake chamber, and simulated and analyzed the static, dynamic and brake chamber pressure response characteristics of the ABS regulator. By analyzing the working principle and parameter test requirements of double spring brake chambers, Sui, L.H. [9] put forward the design scheme of a dynamic performance test system for double spring brake chambers, and completed the whole test platform. Based on different modeling methods for brake chambers, Hu, X.F. [10] proposed a dynamic loading method to establish the brake chamber pressure model and verified the correctness of the model according to the test results. Liang, C. et al. [11] established the joint simulation platform of Matlab (MathWorks.Inc, Natick, MA, USA) and AMESim (SIEMENS AG, Berlin and Munich, Germany), from which the relationship curves between the component parameters of ABS hydraulic control units and the change rates of brake pressure are obtained, and from which the relationship curves between control signals and the physical characteristics of high-speed switching valve are also obtained. Xiaohan, L. et al. [12] established a joint simulation model of pneumatic ABS for commercial vehicles based on AMESim and Simulink software (MathWorks.Inc, Natick, MA, USA), and simulated four typical failure modes of pneumatic ABS to analyze the effects of different failure modes on the braking capacity.

In the development of test systems, Li, Y. [13] designed a test system scheme for non-contact solenoid polarity tests and a solenoid polarity test system based on wireless sensor networks. Feng, J.C. [14] introduced the ICV (Intelligent Connected Vehicle) test system based on virtual simulation technology, semi-physical simulation technology and real-vehicle test technology. Li, D.F. [15] designed the hardware in the loop virtual test system of unmanned vehicles and tested the intelligent behavior of unmanned vehicles in the ways of virtual reality. The system includes four parts: a virtual traffic environment, a road simulation, vehicle dynamics and the simultaneous interpreting of vehicle sensor. It can meet the test requirements of different traffic conditions, different models and different sensors when the driverless vehicle is tested. In order to verify the performance indicators of the test system, Chen, S.M. et al. [16] analyzed the repeatability and reproducibility evaluation indicators of the measurement system using the mean extreme difference calculation method, and proposed a method to improve the repeatability and reproducibility of the measurement system. De Mast, J. et al. [17] studied the standard method of evaluating the accuracy of the measurement system and proposed an alternative method to apply the standard method of evaluating the accuracy of the measurement system in destructive measurement. Hu, Y. et al. [18] proposed a LabVIEW-based LED driver test system. The proposed system was evaluated by Minitab software to ensure its measurement accuracy.

No previous studies have proposed direct measurement methods for the rate of PCR in brake chambers in electronically controlled pneumatic braking systems. The evaluation of the performance indicators of self-developed test systems has mainly focused on the verification of their repeatability and reproducibility, neglecting a comprehensive consideration of test system characteristics such as versatility, stability, repeatability, accuracy and reproducibility. In this paper, the PCR of commercial vehicle brake chambers is studied, and the PCR test system is designed based on theoretical analysis, simulation analysis and experimental verification. The remaining contents of this paper are organized as follows: in Section 2, taking the diaphragm brake chamber as the test object, the PCR threshold of commercial vehicle brake chambers for braking comfort is proposed. In Section 3, the PCR measurement method based on laminar resistance tubes is presented. In Section 4, the PCR test system of commercial vehicle brake chambers is designed. In Section 5, the PCR test system of commercial vehicle brake chambers is simulated and analyzed experimentally. In Section 6, according to the evaluation method of the special testing equipment, the stability, accuracy, repeatability and reproducibility of the test system are evaluated to verify the correctness and applicability of the PCR test system developed in this paper. This research work can provide theoretical support for the precise control of pressure in the braking process of electronically controlled pneumatic braking systems, thus effectively solving the problems of pressure deviation and time deviation in pneumatic braking systems, which is of great significance for the development of intelligent braking.

## 2. Analysis of PCR of Commercial Vehicle Brake Chambers for Braking Comfort

### 2.1. Classification of Commercial Vehicle Brake Chambers

**Figure 1:**
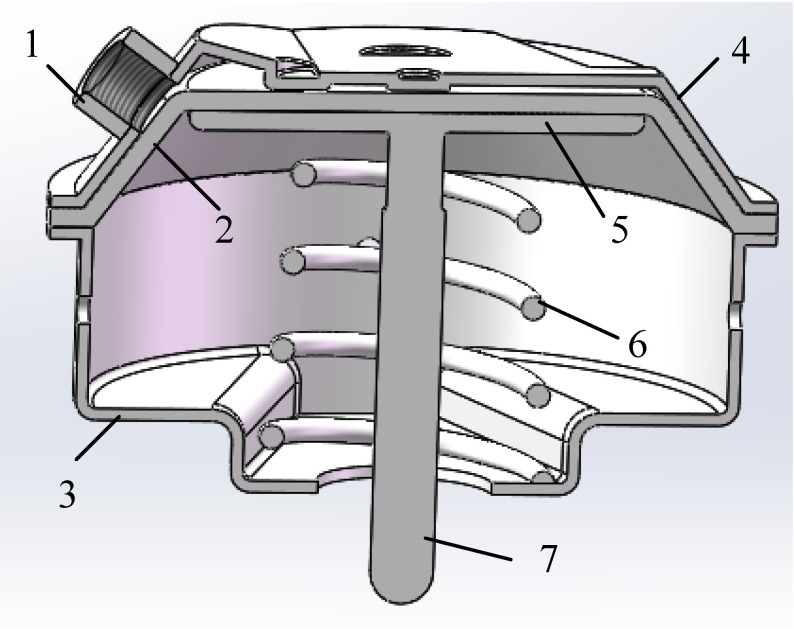
3D profile view of diaphragm brake chamber. 1—Compressed Gas Inlet, 2—Rubber Diaphragm, 3—Lower Cover, 4—Upper Cover, 5—Piston Disc, 6—Return Spring, 7—Pusher.

The brake chambers in the pneumatic braking system of two commercial vehicles are a diaphragm brake chamber and a composite spring brake chamber. The diaphragm brake chamber contains a driving brake chamber controlled by a foot brake valve for vehicle braking. The composite spring brake chamber consists of a driving brake chamber and a parking brake chamber. The driving brake chamber is controlled by a foot brake valve for vehicle braking and the parking brake chamber is controlled by a hand brake valve for parking braking or emergency braking. The diaphragm brake chamber is taken as the research object in this paper. The 3D profile view of the diaphragm brake chamber is shown in Figure 1. The driving brake chamber consists of a rubber diaphragm and a section surrounding the upper cover. When the diaphragm is not moving, the diaphragm is close to the upper cover. When the brake gas chamber is working, the compressed gas pushes the diaphragm to drive the piston disc outward, and the push rod is connected with the brake arm and drives the brake arm outward to complete the braking action.

The brake chamber is the executing part of the commercial vehicle’s electronically controlled pneumatic brake system. The response speed of the brake chamber reflects the performance of the commercial vehicle’s electronically controlled pneumatic brake system. B.2.2 in GB12676-2014 [19] stipulates that when the boost time is 0.2 s, the pressure in the brake chamber will not exceed 0.6 s from the start of the boost to 75% of the maximum steady state value in the brake chamber, and the compressed gas in the brake chamber will be discharged after braking. GB 7258-2017 [20] 7.1.6 stipulates that the full brake release time of two-axle cars should be less than or equal to 0.8 s, The full release time of braking for vehicles with three or more axles shall be less than or equal to 1.2 s.

### 2.2. PCR Threshold of Commercial Vehicle Brake Chambers for Braking Comfort

Intelligent braking requires that the vehicle braking system should not only ensure the safety of the operation process, but also ensure the comfort of the vehicle. Zhang, J.Z. [21] et al. take jerking as the comfort evaluation index, obtain the corresponding relationship between the vehicle jerk and human comfort, as shown in Table 1, to evaluate human comfort during braking.

The vehicle jerk is an evaluation index for the vehicular dynamics. The jerk cannot be controlled in real time through the electronic pneumatic braking system in the process of vehicle braking. Therefore, the jerk is correlated with the performance of the brake chamber, and the corresponding relationship between the vehicle acceleration and the PCR of commercial vehicles brake chamber is:

(1) $$\frac{dp}{dt}=5.288\times 10^{5}\times \frac{da}{dt}$$

where p is the pressure in the inner cavity of the brake air chamber, [Pa]; a is the acceleration of the vehicle, [m/s^2^].

Substituting the threshold value of the evaluation standard in Table 1 into Equation (1), the evaluation standard of occupant comfort with the PCR as the evaluation index is obtained, as shown in Table 2.

## 3. Measurement Method of PCR Based on Laminar Flow Resistance Tubes

**Figure 2:**
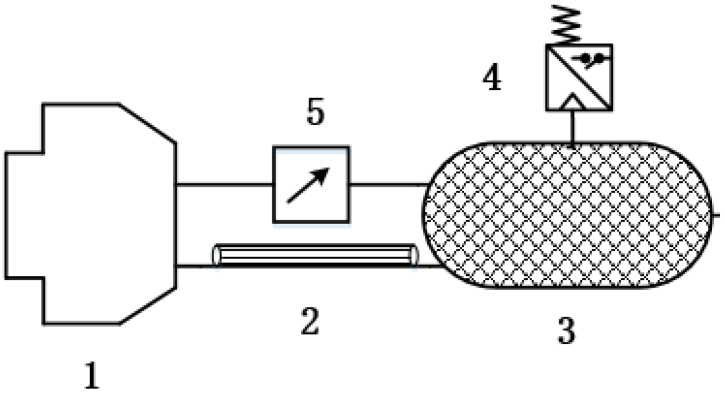
Measurement schematic diagram of PCR based on laminar resistance tubes. 1—Brake Chamber, 2—Laminar Resistance, Tube 3—Isothermal Container, 4—Pressure Sensor, 5—Differential Pressure Sensor.

The measurement principle of PCR for commercial vehicle brake chambers based on laminar flow resistance tubes is shown in Figure 2. It is composed of an isothermal container, a laminar flow resistance tube, a brake chamber, a differential pressure sensor and a pressure sensor. When the commercial vehicle’s electronically controlled pneumatic brake system is working, the brake chamber undergoes the process of charging and deflating, which causes the pressure value $p_{c}$ in the brake chamber to change. Because the brake chamber is connected to the isothermal container through a laminar resistance tube, which acts as a flow resistance through its gas, the change of $p_{c2}$ in the isothermal container lags behind, resulting in a pressure difference $p_{j2}$ between the brake chamber and the isothermal container. $p_{j2}$ is measured by a pressure difference sensor, and $p_{c2}$ in the isothermal container is measured by a pressure sensor. Based on Hagen–Poiseuille’s law and gas state equation, the relationship between PCR $dp_{c2}/dt$ and $p_{j2}$, $p_{c2}$ in the isothermal container is deduced. The measurement value of PCR $dp_{c2}/dt$ in the brake chamber can be approximated by the PCR $dp_{s}/dt$ in the isothermal container, so as to achieve the measurement of PCR for commercial vehicle brake chambers.

It is assumed that the flow in the resistance tube is laminar. The state equation of gas in the isothermal container is:

(2) $$p_{c2}V=WR\theta$$

By full differential:

(3) $$V\frac{dp_{c2}}{dt}+p_{c2}\frac{dV}{dt}=GR\theta +WR\frac{d\theta }{dt}$$

Because of the volume of isothermal in the container, Formula (3) can be changed into:

(4) $$G=\frac{V}{R\theta_{a}}\frac{dp_{c2}}{dt}$$

where V is the volume of the isothermal container, [$m^{3}$]; R is the gas constant, [J/(kg·K)]; W is the gas mass inside the isothermal container, [kilograms]; is the internal temperature of the isothermal container, [K]; $\theta_{a}$ is the ambient temperature, [K]; is the gas mass flow G through the laminar resistance tube in the pneumatic circuit, which is proportional to the PCR $dp_{c2}/dt$ in the isothermal container.

Set the number of capillaries in the laminar flow resistance tube as n, the inner diameter of the capillaries as r and the length as L. According to Poiseuille’s law, the gas volume flow into the isothermal container is:

(5) $$Q=\frac{\pi nr^{4}}{8\mu L}\left(p_{s2}-p_{c2}\right)$$

By $\frac{p_{c2}}{p_{a}}=\frac{\rho_{c2}}{\rho_{a}}$, G through the laminar resistance tube is:

(6) $$G=\rho_{c2}Q=\frac{\rho_{a}p_{c2}\pi nr^{4}}{8p_{a}\mu L}\left(p_{s}-p_{c2}\right)$$

where $p_{a}$ is atmospheric pressure, [Pa]; $\rho_{a}$ is the density of the atmosphere, [$kg/m^{3}$]; $\rho_{c2}$ is the gas density inside the isothermal container, [$kg/m^{3}$]; is the aerodynamic viscosity coefficient, [Pa⋅s].

When the flow resistance coefficient of the laminar resistance tube is set to $r_{s}=\frac{8p_{a}\mu L}{\rho_{a}\pi nr^{4}}$, the Substitution (6) is:

(7) $$G=\frac{p_{c2}}{r_{s}}\left(p_{s}-p_{c2}\right)$$

The output of the pressure difference sensor is:

(8) $$p_{j2}=p_{s}-p_{c2}$$

Based on the Formulas (4), (7) and (8), the PCR mathematical model of the commercial vehicle’s brake chamber can be obtained as follows:

(9) $$\frac{dp_{c2}}{dt}=\frac{p_{c2}p_{j2}}{r_{s}}\frac{R\theta_{a}}{V}$$

## 4. Design of PCR Test System for Commercial Vehicle Brake Chambers

### 4.1. Overall Scheme of PCR Test System for Commercial Vehicle Brake Chambers

**Figure 3:**
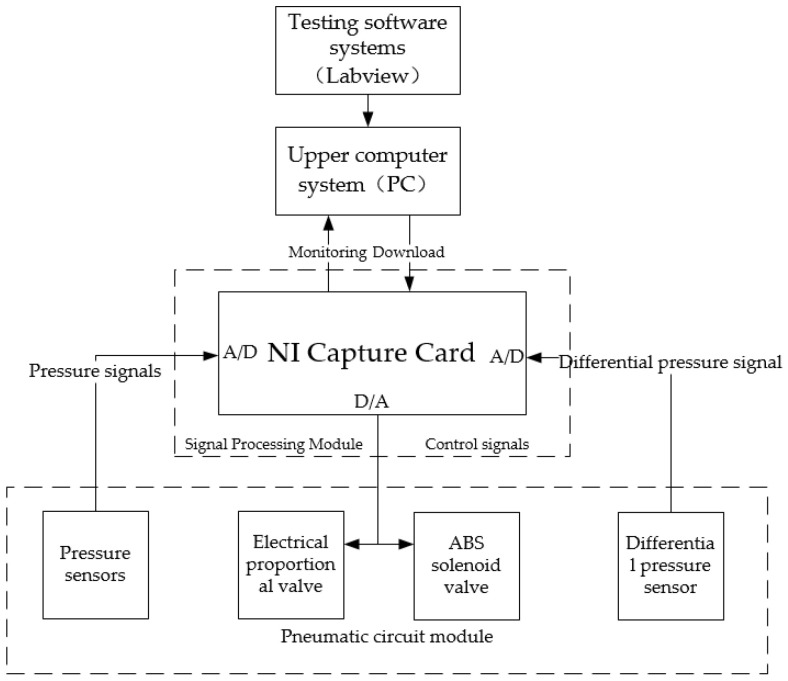
Overall structure scheme of PCR test systems for commercial vehicle brake chambers.

The PCR test system for commercial vehicle brake chambers consists of four parts: a pneumatic circuit module, a signal processing module, an upper computer system and a test software system. The overall structure scheme of PCR test systems for commercial vehicle brake chambers is shown in Figure 3.

**Figure 4:**
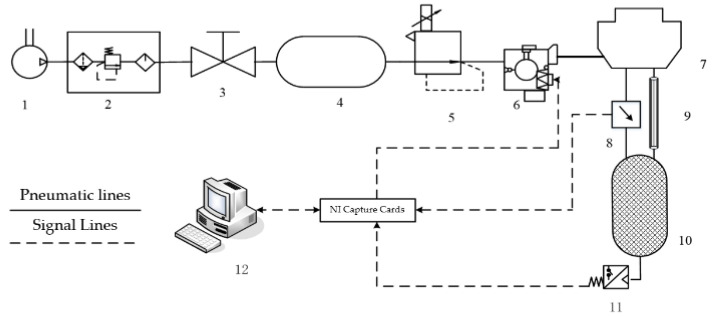
Pneumatic circuit diagram of the PCR test system for brake chambers. 1—air source, 2—pneumatic triplet, 3—on–off valve, 4—air reservoir, 5—electrical proportional valve, 6—ABS solenoid valve, 7—brake air chamber, 8—differential pressure sensor, 9—laminar flow resistance tube, 10—isothermal container, 11—pressure sensor, 12—industrial computer.

The pneumatic circuit module is the core module of the test system, mainly including an air source module, an electrical equipment module, a circuit control module and a test circuit module. The pneumatic circuit diagram of the PCR test system is shown in Figure 4.

The signal processing module is in charge of collecting and processing the digital signals and output control signals generated by the working of the test system, and is also in charge of realizing the real-time communication of the test system. The signal processing module mainly includes the collecting and processing of pressure sensor signals and differential pressure sensor signals, the control signal outputs of the electric proportional valve and ABS solenoid valve, and the storage of the test data.

The upper computer sends control signals to the acquisition card, which controls the electrical proportional valve, ABS solenoid valve and other components according to the signal. The NI acquisition card reads the sensor signal, converts it into digital signal and feeds it back to the upper computer for data processing and analysis, and outputs the results.

The software system is developed by the graphical programming language LabVIEW (National Instruments, Austin, TX, USA), which mainly includes the test system’s control program and user interface. The test system’s control program can not only send control signals to realize the control of the ABS solenoid valve and electrical proportional valve, but also collect the pressure signals of the pressure sensor and differential pressure sensor The signal is processed to obtain the PCR of the brake chamber. The user interface is in charge of the real-time operation of the test system and allows for test operation and data management for the user.

### 4.2. Hardware Design of PCR Test System for Commercial Vehicle Brake Chambers

According to the pneumatic circuit scheme of the test system, the required components are selected as shown in Table 3.

According to the measurement principle of PCR based on the laminar flow resistance tube, it is necessary to design and manufacture the brake chamber, laminar flow resistance tube and isothermal container.

A diaphragm brake chamber with type C3519D010A is selected in this paper. The C3519D010A diaphragm brake chamber is selected to drill holes on the inner and outer surfaces of the chamber for installing threaded pipe connections. The upper threaded pipe connection is connected to the laminar resistance pipe, and the lower threaded pipe connection is connected to the differential pressure sensor. A certain number of capillaries are connected in parallel to form a laminar resistance tube. Based on the simulation method adopted by Yang, R. [22], the size parameters of the laminar resistance tube are shown in Table 4. The isothermal container consists of a vessel and filler. The vessel is a container with a diameter d = 50 mm, height h = 20 mm and volume $V=4\times 10^{-5}m^{3}$. The filler is copper wire with a diameter of 50 μm and filling density of $300\;kg/m^{3}$.

**Figure 5:**
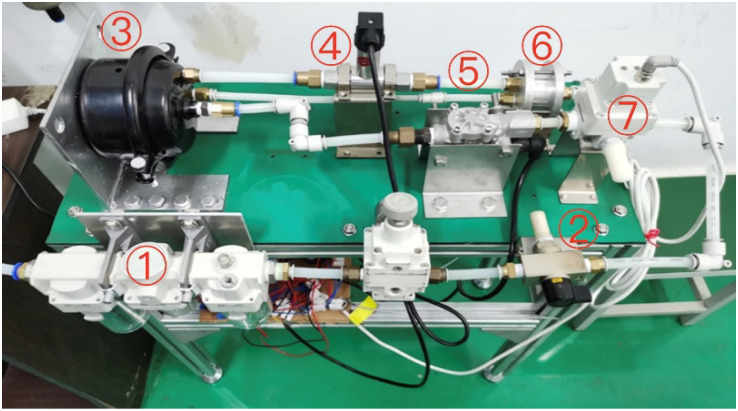
Hardware test system of PCR for commercial vehicle brake chambers. 1—pneumatic triplet, 2—on–off valve, 3—air reservoir, 4—differential pressure sensor, 5—laminar flow resistance tube, 6—isothermal container, 7—electrical proportional valve.

The two D/A converter outputs of the NI acquisition card are connected to an electrical proportional valve and an ABS solenoid valve. The two A/D converter inputs are connected to an isothermal vessel pressure sensor and a differential pressure sensor. The hardware test system of PCR for commercial vehicle brake chambers is shown in Figure 5.

### 4.3. Software Design of PCR Test System for Commercial Vehicle Brake Chambers

The LabVIEW programming language is used to develop the software program of the PCR test system. The data acquisition card collects the signals from the pressure sensor and differential pressure sensor, and uses the driver and LabVIEW test system software to transfer the data. The LabVIEW program outputs the control signals to control the electrical proportional valve and ABS valve to complete the PCR measurement of the brake chamber and display the test results on the test system’s software interface.

**Figure 6:**
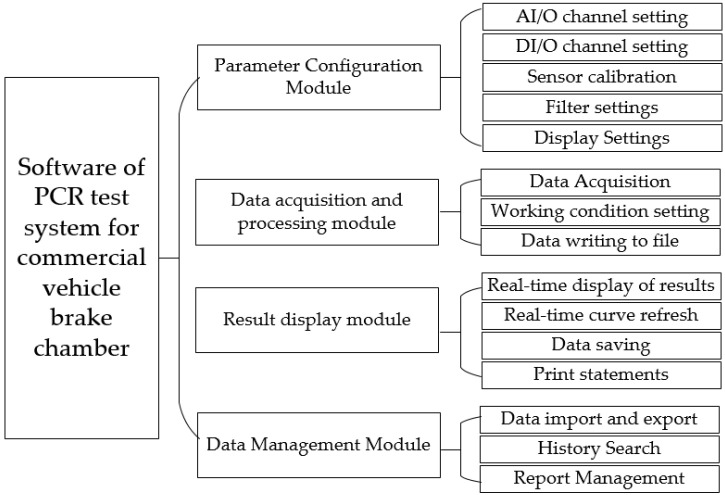
Software module of the PCR test system for commercial vehicle brake chambers.

The software of the PCR test system for commercial vehicle brake chambers is developed by using the modular programming method. The system includes a parameter configuration module, data acquisition and processing module, result display module and data management module. The modules and their functions are shown in Figure 6.

## 5. Simulation and Experimental Analysis of PCR Test System for Commercial Vehicle Brake Chambers

### 5.1. Simulation Model of PCR Test System for Commercial Vehicle Brake Chambers

#### 5.1.1. Model of Electrical Proportional Valve

The mathematical model of the electrical proportional valve consists of three parts: the electrical proportional valve orifice flow equation, the gas flow continuity equation and the spool force balance equation. The equation for the mass flow rate G of the gas discharged from the electrical proportional valve is:

(10) $$G=c_{q}A_{q}p_{r}\sqrt{\frac{2}{R\theta }}\varphi \left(\frac{p_{c}}{p_{r}}\right)=c_{q}Wx_{v}p_{r}\sqrt{\frac{2}{R\theta }}\varphi \left(\frac{p_{c}}{p_{r}}\right)$$

(11) $$\varphi \left(\frac{p_{c}}{p_{r}}\right)=\left\{\begin{array}{cc} \sqrt{\frac{r}{r-1}\left[{\left(\frac{p_{c}}{p_{r}}\right)}^{\frac{2}{r}}-{\left(\frac{p_{c}}{p_{r}}\right)}^{\frac{r+1}{r}}\right]} & 0.528\leq \left(\frac{p_{c}}{p_{r}}\right)\leq 1\\ {\left(\frac{2}{r+1}\right)}^{\frac{r}{r-1}}\sqrt{\frac{r}{r+1}} & 0\leq \left(\frac{p_{c}}{p_{r}}\right)\leq 0.528 \end{array}\right.$$

where $c_{q}$ is the gas flow coefficient; $A_{q}$ is the effective open area of the spool; R is the gas constant; θ is the gas temperature; $p_{c}$ is the pressure at the exhaust port of the electrical proportional valve; $p_{r}$ is the pressure at the inlet port of the electrical proportional valve; $x_{v}$ is the displacement of the electrical proportional valve spool; r is the specific heat ratio of the gas.

The gas mass flow rate into the load chamber is:

(12) $$G=\frac{V}{rRT}\frac{dp_{c}}{dt}$$

The equation for the equilibrium of forces on the valve spool is:

(13) $$m\frac{d^{2}x_{v}}{dt^{2}}=p_{1}A_{1}-p_{v}A_{2}-k_{f}(x_{v}+x_{0})-f-c\frac{dx_{v}}{dt},$$

where m is the mass of the spool, [kg]; $p_{1}$ is the pilot gas chamber pressure, [Mpa]; $A_{1}$ is the effective area at the top of the electrical proportional valve stem, [$m^{2}$]; $A_{2}$ is the effective area at the bottom of the electrical proportional valve stem, [$m^{2}$]; $k_{f}$ is the spool’s spring stiffness, [N/m]; $x_{0}$ is the pre-compression of the spool spring, [m]; f is the motion friction, [N]; and c is the valve stem motion damping coefficient, [N/(m/s)].

**Figure 7:**
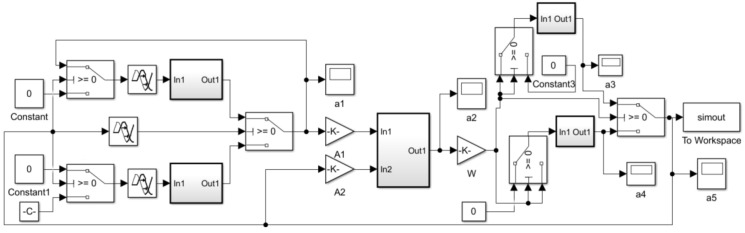
Simulation model (Simulink model) of the electrical proportional valve.

The simulation model (Simulink model) based on the mathematical model of the electrical proportional valve is shown in Figure 7.

The key parameters for the simulation of the electrical proportional valve are shown in Table 5.

#### 5.1.2. Model of Laminar Flow Resistance Tube

The differential pressure ΔP between the left and right ends of the laminar flow resistance tube is:

(14) $$\Delta P=K_{1}\mu Q+K_{2}\rho Q^{2}$$

where Q is the volume flow of gas through the laminar resistance tube, [m^3^/h]; μ is the gas dynamic viscosity; ρ is the gas density; K_1_, K_2_ are the laminar resistance tube structure size coefficients.

**Figure 8:**
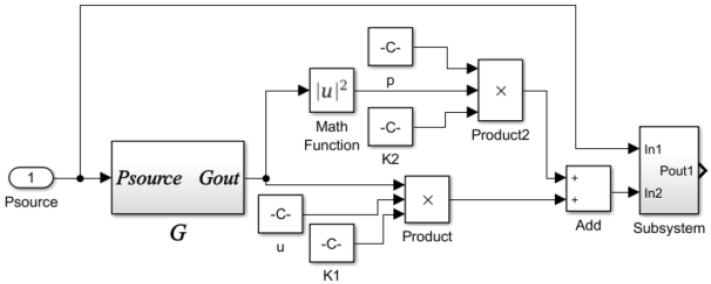
Laminar flow resistance tube simulation model (Simulink model).

The simulation model (Simulink model) of the laminar flow resistance tube based on the mathematical model is shown in Figure 8.

#### 5.1.3. Model of Isothermal Vessel

The isothermal vessel can ensure that the gas in the vessel is approximating an isothermal process during the filling and discharging of gas. The mass flow out of the isothermal vessel can be deduced by measuring the pressure when the isothermal vessel is deflated. The isothermal vessel’s mathematical model can be composed of the following parts.

The gas state equation in differential form is:

(15) $$\frac{dp}{dt}=\frac{1}{V_{d}}\left(RT\frac{dm_{d}}{dt}+\frac{pV_{d}}{T}\frac{dT}{dt}\right)$$

where p is the absolute pressure of the gas, [MPa]; $V_{d}$ is the volume of the isothermal container, [$m^{3}$]; $m_{d}$ is the mass of the gas in the isothermal container, [kg]; T is the absolute temperature of the gas, [K].

The energy equation is:

(16) $$\frac{dT}{dt}=\frac{1}{c_{v}m_{d}}\left[-GRT+TF(T_{s}-T)\right]$$

where T is the convective heat transfer coefficient; F is the heat transfer area, [m^2^]; T_s_ is the absolute temperature of the filling material, [K].

The internal filling material heat transfer equation is:

(17) $$\frac{dT_{s}}{dt}=-\frac{1}{c_{s}m_{s}}\frac{dQ}{dt}$$

where c_s_ is the specific heat capacity of the filling material; m_s_ is the mass of the filling material, [kg].

The convective heat transfer equation is:

(18) $$\frac{dQ}{dt}=TF\left(T_{s}-T\right)$$

The flow equation is:

(19) $$G=-\frac{dm_{d}}{dt}=\left\{\begin{array}{cc} C\rho_{0}p\sqrt{\frac{T_{0}}{T}} & 0<\frac{p_{a}}{p}\leq b\\ C\rho_{0}p\sqrt{\frac{T_{0}}{T}}\sqrt{1-\left(\frac{p_{a}/p-b}{1-b}\right)} & b\leq \frac{p_{a}}{p}\leq 1 \end{array}\right.$$

where C is the sonic flow conductance; $\rho_{0}$ is the gas density in a standard state, [$kg/m^{3}$]; $T_{0}$ is the gas temperature in a standard state, [K]; $p_{a}$ is the pressure of the filling material, [MPa]; b is the critical pressure ratio.

**Figure 9:**
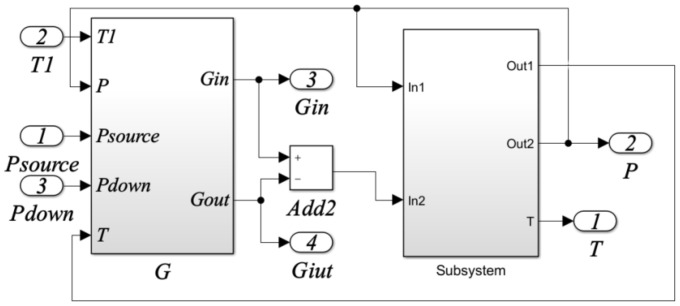
Isothermal vessel simulation model (Simulink model).

The simulation model (Simulink model) of the isothermal vessel based on the mathematical model is shown in Figure 9.

**Figure 10:**
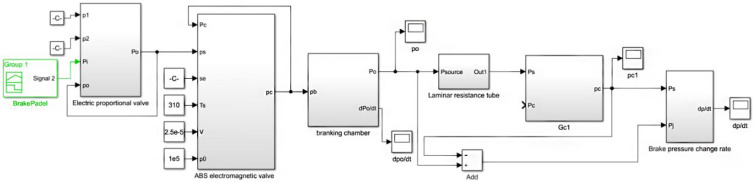
Simulation model (Simulink model) of the PCR test system for commercial vehicle brake chamber.

According to the pneumatic circuit and working principle of the test system, the simulation model (Simulink model) of the PCR test system for commercial vehicle brake chambers is established as shown in Figure 10. The simulation model includes the electrical proportional valve, ABS valve, brake air chamber, laminar flow resistance tube, isothermal container and the corresponding piping models.

### 5.2. Simulation Analysis of PCR Test System for Commercial Vehicle Brake Chambers

Based on the simulation model of the PCR test system for commercial vehicle brake chambers, the pressure rise and drop processes of the commercial vehicle brake chamber are simulated. The PCR curve of the brake chamber is obtained, and its change rule is analyzed.

#### 5.2.1. PCR of Commercial Vehicle Brake Chambers during Pressurization

**Figure 11:**
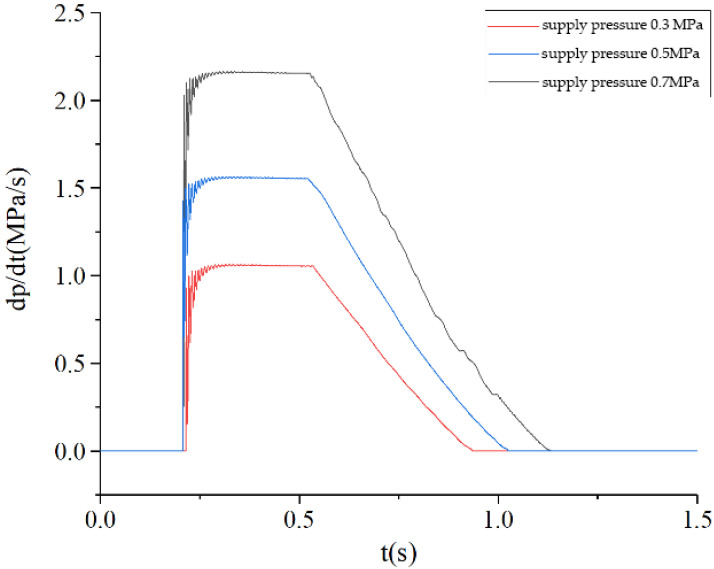
PCR curve during brake chamber pressurization.

The air source pressure is set to 0.7 MPa and the air supply pressure is set to 0.3 MPa, 0.5 MPa and 0.7 MPa, respectively. The inlet valve of the ABS solenoid valve is set to remain open and the exhaust valve is set to remain closed. The pressure rise process of the commercial vehicle brake chamber is simulated, and the PCR curve of the brake chamber is obtained as shown in Figure 11. The PCR change rule of the brake chamber is consistent in the process of inflation under different air supply pressures. The higher the supply pressure, the higher the PCR peak of the brake chamber, and the more obvious the fluctuation of the PCR at the initial stage of the brake chamber’s inflation. The response time of the PCR of the brake chamber is not related to the initial supply pressure.

#### 5.2.2. PCR of Commercial Vehicle Brake Chambers during Depressurization

**Figure 12:**
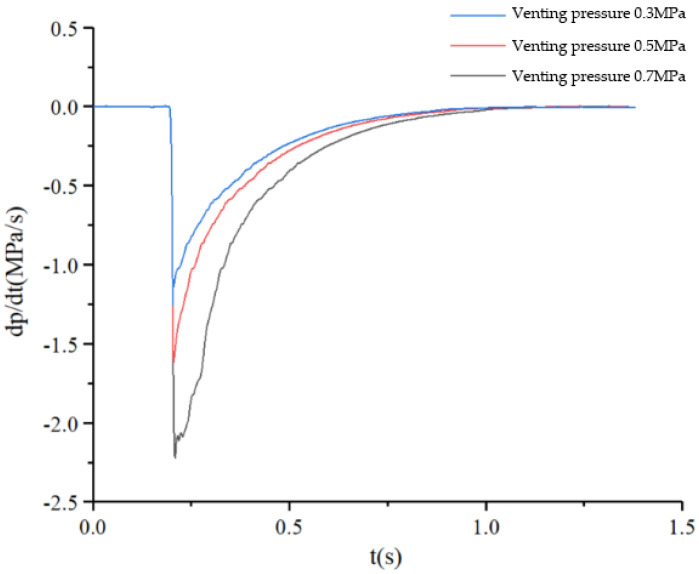
PCR curve during brake chamber depressurization.

The initial pressure inside the commercial vehicle brake chamber is set to 0.3 MPa, 0.5 MPa and 0.7 MPa respectively. The inlet valve of the ABS solenoid valve is set to remain closed, and the exhaust valve is set to remain open. The pressure drop process of the commercial vehicle brake chamber is simulated, and the PCR curve of the brake chamber is obtained as shown in Figure 12. The PCR change rule of the brake chamber is consistent in the process of bleeding at different initial pressures. At the beginning of the brake chamber’s bleeding, there is a time delay for the pressure drop, and the PCR is 0. Then, the pressure inside the brake chamber starts to drop, and the absolute value of the PCR increases rapidly to the maximum value. When the pressure inside the brake chamber is completely discharged, the PCR of the brake chamber becomes 0.

### 5.3. Experimental Analysis of PCR Test System for Commercial Vehicle Brake Chambesr

**Figure 13:**
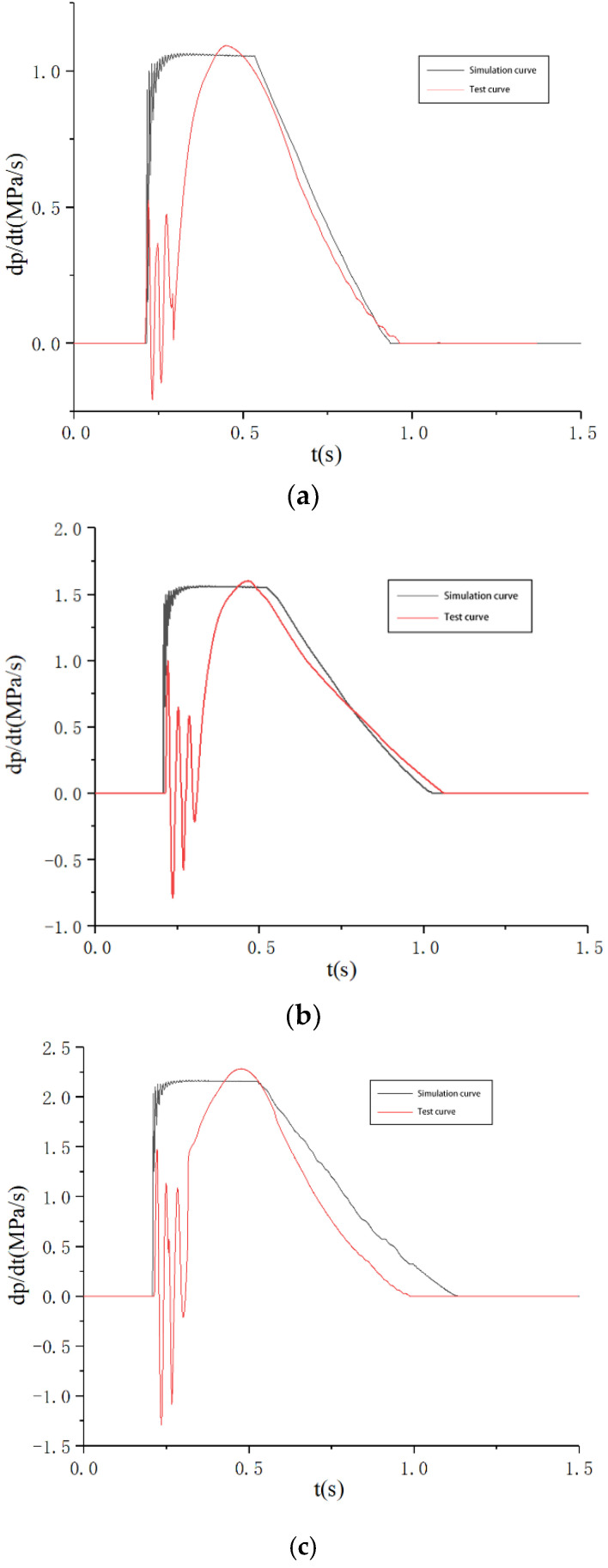
Test curves of PCR during pressurization. (**a**) 0.3 MPa, (**b**) 0.5 MPa, (**c**) 0.7 MPa.

In order to investigate the PCR of commercial vehicle brake chambers under different braking intensities from a non-braking state, the experimental test was conducted with 0.3 MPa, 0.5 MPa and 0.7 MPa to represent three different braking intensities with light-intensity braking, medium-intensity braking and emergency braking, respectively. The air supply pressure is set to 0.7 MPa, and the target pressures are 0.3 MPa, 0.5 MPa and 0.7 MPa. The comparison between the simulation results and experimental results of the PCR test system is shown in Figure 13.

There are some deviations between the PCR test curve and the PCR simulation curve due to various errors during the test system’s operation, but the PCR test curve change rule of the brake chamber is consistent with the simulation curve change rule during pressurization. At the beginning of the brake chamber inflation, the compressed gas enters the brake chamber rapidly, resulting in a sharp increase in the pressure of the brake chamber, so the PCR of the brake chamber increases sharply, and there are violent fluctuations. During the increasing process for the pressure of the brake chamber, the brake chamber push rod moves outward continuously, and the PCR increases to the maximum value and then remains slightly fluctuating around the maximum value. At the last phase of the brake chamber’s inflation, the brake chamber push rod gradually reaches the full stroke, and the PCR gradually decreases. At the end of the brake chamber’s inflation, the pressure of the brake chamber reaches the maximum value and remains unchanged, and the PCR decreases to 0.

**Figure 14:**
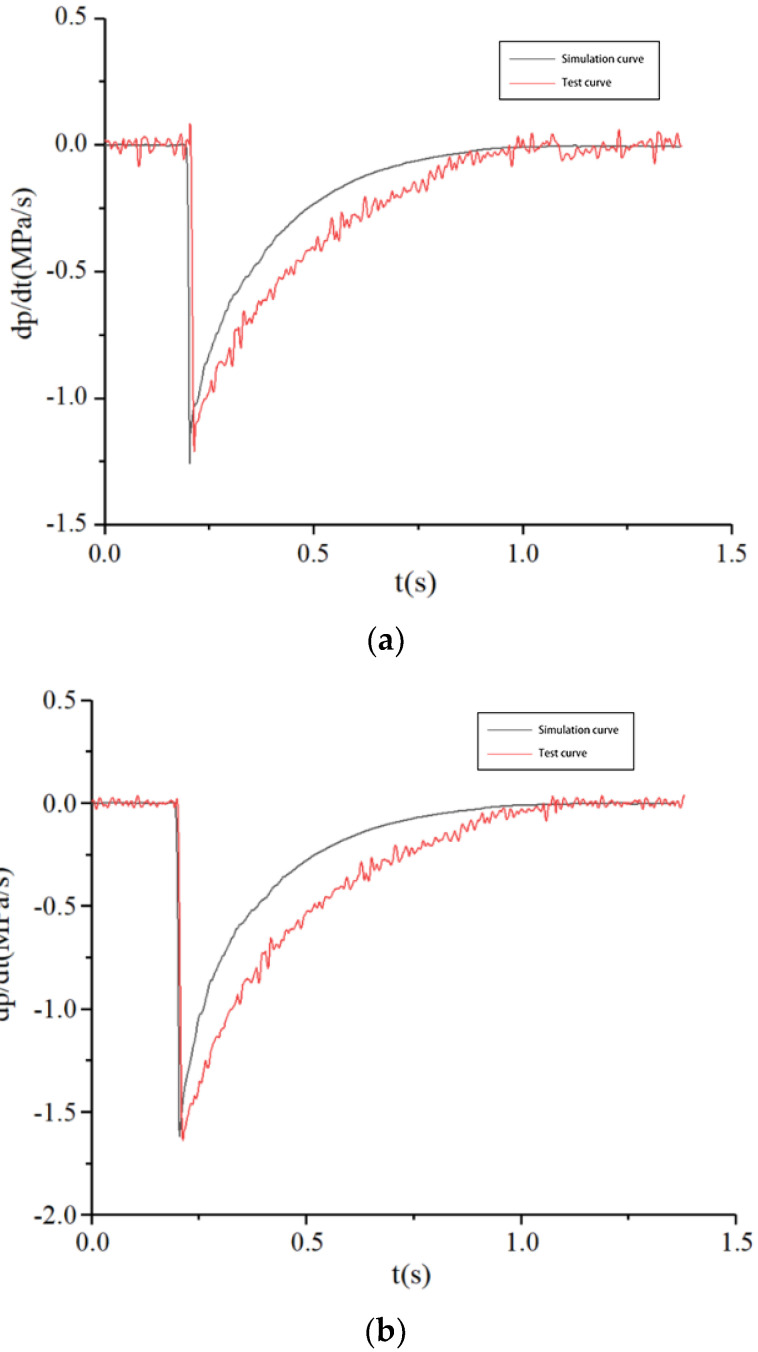

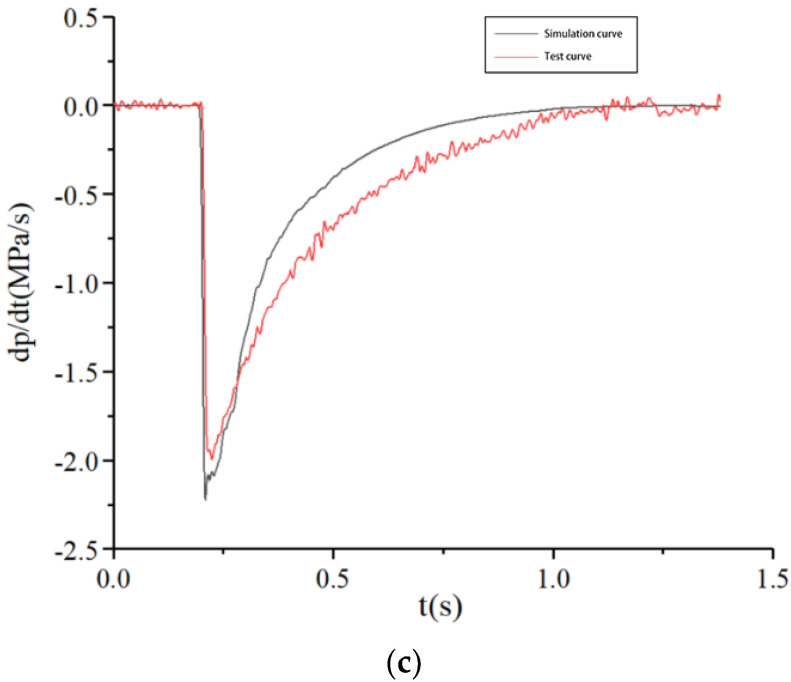
Test curves of PCR during depressurization. (**a**) 0.3 MPa, (**b**) 0.5 MPa, (**c**) 0.7 MPa.

In order to study the change rule of the PCR of the brake chamber during depressurization in the braking release process of commercial vehicles, the PCR of the brake chamber with the pressure of 0.3 MPa, 0.5 MPa and 0.7 MPa are tested. The comparison between the simulation results and experimental results of the PCR test system is shown in Figure 14.

The PCR test curve change rule of the brake chamber is consistent with the simulation curve change rule during depressurization. At the beginning of the depressurization, there is a time delay zone, the pressure of the brake chamber at this stage still maintains the initial value and the PCR is 0. The depressurization stage is different from the pressurization stage, as the PCR of brake chamber is negative and the absolute value of the PCR at the beginning of the depressurization rapidly reaches the maximum and then drops rapidly. When the pressure of the brake chamber is completely discharged, the PCR becomes 0.

According to the test results, the PCR of the brake chamber is less than 2.6 MPa/s during the braking process and the unbraking process, which meets the PCR threshold requirement of commercial vehicle brake chambers and achieves the functional requirements of the test system. The PCR test curves of the commercial vehicle brake chamber in the process of pressurization and depressurization are consistent with the simulation curves, which verified the correctness of the PCR test system for commercial vehicle brake chambers designed in this paper.

## 6. Performance Verification of PCR Test System for Commercial Vehicle Brake Chambers

In order to verify the performance of the PCR test system for commercial vehicle brake chambers according to the test system’s standards, the MSA (Measurement System Analysis) method is applied to analyze the test system’s performance indicators. In this paper, the MSA method is used to verify the stability, accuracy, repeatability and reproducibility of the test system.

### 6.1. Stability Verification of PCR Test System for Commercial Vehicle Brake Chambers

The stability verification steps of the PCR test system for commercial vehicle brake chambers are as follow:

(1) The test system parameters are configured and the standard values of test results are determined.
(2) Test three to five times at the same moment under the same conditions and record the test data in chronological order.
(3) After completing the cycle test, enter all test data into the Minitab table, calculate the control limits and plot the mean–standard deviation $\bar{X}-S$ graph.
(4) According to the $\bar{X}-S$ graph, if the stability of the test system meets the requirements, the report will be recorded, and the stability analysis of the test system will be completed. If the stability of the test system does not meet the requirements, the reasons for the poor stability will be analyzed and improved, and the stability of the test system will be determined again.

**Figure 15:**
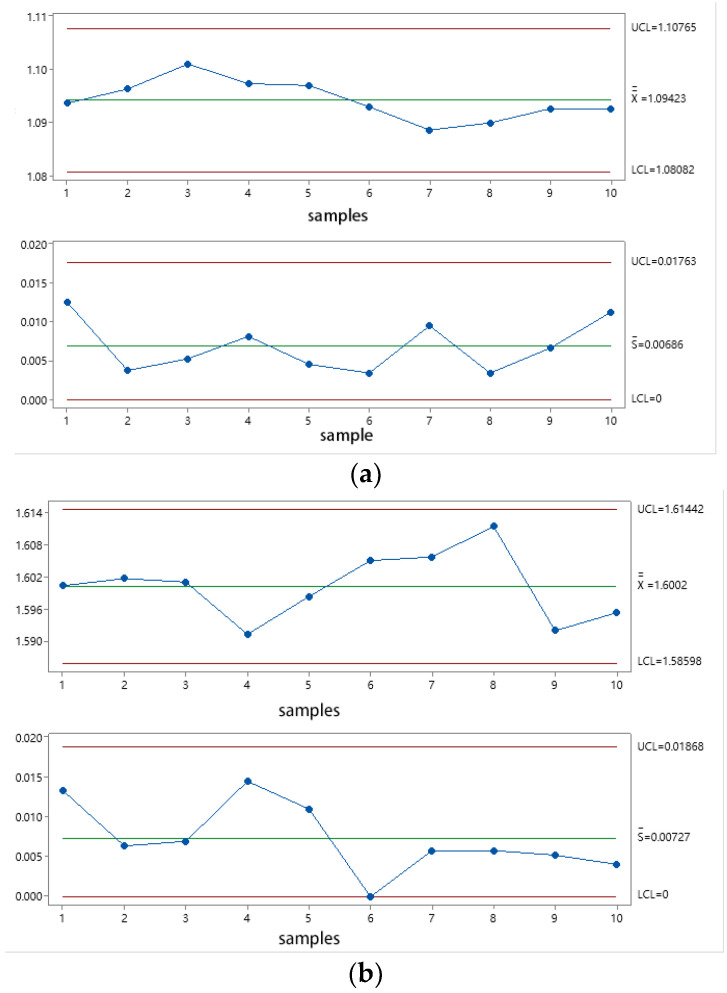

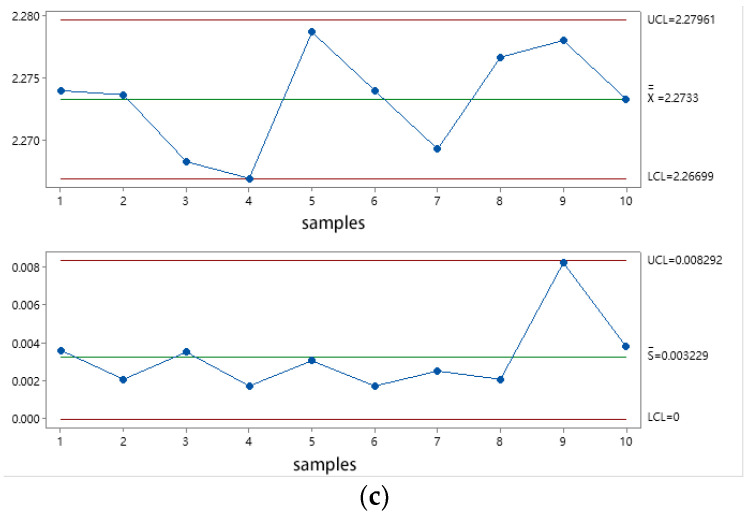
The mean–standard deviation $\bar{X}-S$ control chart of the PCR test system for commercial vehicle brake chamber. (**a**) 0.3 MPa $\bar{X}-S$, (**b**) 0.5 MPa $\bar{X}-S$, (**c**) 0.7 MPa $\bar{X}-S$.

In order to evaluate the stability of the PCR test system for commercial vehicle brake chambers, the PCR of the brake chamber is tested 3 times regularly (9:00 a.m. every day) for 10 consecutive days, and 3 boost conditions of 0.3 MPa, 0.5 MPa and 0.7 MPa are selected. All test data from the test system are processed using Minitab to calculate the mean $\bar{X}$, extreme deviation R and standard deviation S of the test data, as well as the $\bar{\bar{X}}$ (the mean of mean $\bar{X}$), the $\bar{S}$ (the mean of the standard deviation S) and the upper and lower limits of the mean and standard deviation. The mean–standard deviation $\bar{X}-S$ control chart of the PCR test system for commercial vehicle brake chambers is shown in Figure 15. The mean values and standard deviations of the test data during pressurization with target pressures 0.3 MPa, 0.5 MPa and 0.7 MPa are all between the permissible upper and lower limits, in line with the test system’s stability evaluation index, and the test system stability is acceptable.

### 6.2. Accuracy Verification of PCR Test System for Commercial Vehicle Brake Chambers

The accuracy verification steps of the PCR test system for commercial vehicle brake chambers are as follow:

(1) Data collection: test the PCR of the brake chamber for n times in succession at very short intervals and record the value Xi for each test. Before each test, the components such as the brake chamber must not be changed and the testers must be the same person.
(2) Calculating the standard deviation S_g_: calculate the average value $\bar{X_{g}}$ based on the data values Xi with n times tests, and substitute each test value Xi and the average value into the standard deviation formula of the test results to obtain the standard deviation S_g_.
(3) Calculating the offset Bi: Substitute the reference value Xm and the average value $\bar{X_{g}}$ of the test results into the formula for the absolute value of the difference between the average value of the test system test and the reference value of the test to obtain the offset Bi.
(4) Calculating the accuracy index C_gk_: Substitute the tolerance T, standard deviation S_g_ and offset Bi into the measurement capability index formula to obtain the accuracy index C_gk_ of the test system.

Because the brake chamber is a braking safety component, the accuracy index of the PCR test system for the commercial vehicle brake chamber should meet $C_{gk}\geq 1.67$. The PCR of the brake chamber is tested 30 times without interruption for a specified period. The target pressures for pressurization with 0.3 MPa, 0.5 MPa and 0.7 MPa are selected for the test.

(1) The PCR measurement results of the 0~0.3 MPa pressurization phase: the average measurement value $\bar{X_{g1}}$ is 1.0939 MPa/s, the measurement tolerance T is 2.6 MPa/s and the standard deviation S_g1_ is 0.00723 MPa/s. According to the accuracy index calculation method, the accuracy index C_gk_ of the test system is 2.31, which is greater than 1.67, meeting the requirements for a qualified decision.
(2) The PCR measurement results of the 0~0.5 MPa pressurization phase: the average measurement value $\bar{X_{g1}}$ is 1.6042 MPa/s, the measurement tolerance T is 2.6 MPa/s and the standard deviation S_g1_ is 0.00721 MPa/s. According to the accuracy index calculation method, the accuracy index C_gk_ of the test system is 2.87, which is greater than 1.67, meeting the requirements for a qualified decision.
(3) The PCR measurement results of the 0~0.7 MPa pressurization phase: the average measurement value $\bar{X_{g1}}$ is 2.2738 MPa/s, the measurement tolerance T is 2.6 MPa/s and the standard deviation S_g1_ is 0.00785 MPa/s. According to the accuracy index calculation method, the accuracy index C_gk_ of the test system is 3.34, which is greater than 1.67, meeting the requirements for a qualified decision.

Therefore, the PCR test system for commercial vehicle brake chambers meets the accuracy index.

### 6.3. Repeatability and Reproducibility Verification of PCR Test System for Commercial Vehicle Brake Chambers

The “GRR method” is used to verify the repeatability and reproducibility of the PCR test system for commercial vehicle brake chambers in this paper. The evaluation indicator “GRR” reflects the repeatability and reproducibility of the test system and is expressed as a percentage of the total process error of the test in %GRR, which directly reflects whether the test system is qualified. It is shown that if the %GRR is less than 10%, the test system is considered to meet the repeatability and reproducibility criteria, and the test system passes. If the %GRR is between 10% and 30%, the test system is considered to basically meet the repeatability and reproducibility criteria, and the test system is acceptable in some applications. If the %GRR is greater than 30%, the test system is considered to not meet the repeatability and reproducibility criteria, and the test system fails. Three testers are selected, A, B and C. Ten brake chambers are selected and numbered (1,2...10), and each person tests all brake chambers three times respectively. The extreme difference $R_{i}$, mean extreme difference $\bar{R_{i}}$, total mean extreme difference $\bar{\bar{R}}$, total mean value $\bar{\bar{X}}$ and mean maximum difference ${\bar{X}}_{diff}$ of the test data are calculated, and the repeatability–reproducibility index GRR of the test system is deduced.

(1) Repeatability and reproducibility evaluation of the test system with a pressurization target pressure of 0.3 MPa: ${\bar{\bar{R}}}_{1}$ is 0.00337 MPa, ${\bar{X}}_{diff1}$ is 0.00133 MPa and the total variation TV is 0.10, so the repeatability–equipment variation EV is 0.01027, and the reproducibility-tester variation AV is 0.00307. The repeatability–reproducibility index %GRR is 7.15%, which is less than 10%, so the repeatability–reproducibility of the test system with a pressurization target pressure of 0.3 MPa is acceptable and meets the requirements.
(2) Repeatability and reproducibility evaluation of test system with pressurization target pressure of 0.5 MPa: ${\bar{\bar{R}}}_{1}$ is 0.00563 MPa, ${\bar{X}}_{diff1}$ is 0.00290 MPa and the total variation TV is 0.15, so the repeatability–equipment variation EV is 0.01718, and the reproducibility-tester variation AV is 0.00717. The repeatability–reproducibility index %GRR is 6.65%, which is less than 10%, so the repeatability–reproducibility of the test system with a pressurization target pressure of 0.5 MPa is acceptable and meets the requirements.
(3) Repeatability and reproducibility evaluation of the test system with a pressurization target pressure of 0.7 MPa: ${\bar{\bar{R}}}_{1}$ is 0.00833 MPa, ${\bar{X}}_{diff1}$ is 0.00231 MPa and the total variation TV is 0.22. So, the repeatability–equipment variation EV is 0.02542, and the reproducibility–tester variation AV is 0.00622. The repeatability–reproducibility index %GRR is 2.58%, which is less than 10%, so the repeatability–reproducibility of the test system with a pressurization target pressure of 0.7 MPa is acceptable and meets the requirements.

Based on the results of the repeatability–reproducibility assessment of the test system, the %GRR index with three different target pressures is less than 10%. The PCR test system for commercial vehicle brake chambers is considered acceptable.

## 7. Conclusions

In order to comprehensively consider the pressure response and time response of the brake chamber in the braking process and realize the accurate control of intelligent braking and the comfort control of commercial vehicles, the PCR threshold value of commercial vehicle brake chambers for brake comfort and the PCR measurement method based on laminar flow resistance tube are proposed. The overall scheme of the PCR test system for commercial vehicle brake chambers is designed, and the hardware and software of the test system are developed. The mathematical model of each component is established, and the simulation model of the PCR test system for commercial vehicle brake chambers is constructed. The correctness of the test system is verified by simulation analysis and experimental results. The performance of the test system is verified by an MSA analysis method.

## Figures and Tables

**Table 1:** Evaluation criteria of occupant comfort based on jerking.

Jerk (m/s^3^)	Below 5.0	5.0~10.0	Over 10.0
Evaluation grade	Comfortable	Relatively comfortable	Uncomfortable

**Table 2:** Evaluation criteria of occupant comfort based on PCR.

PCR (MPa/s)	Below 2.6	2.6~5.2	Over 2.5
Evaluation grade	Comfortable	Relatively comfortable	Uncomfortable

**Table 3:** Key components of PCR test system for commercial vehicle brake chamber.

Components	Model	Parameter	Quantity
Pneumatic triplet	AC20-A	Interface diameter: 12 mm	1
Manual on–off valve	VHK3-06F-06F	Working pressure range: 0~1.0 MPa	1
Precision pressure-reducing valve	IR1020-01	Pressure-regulating range: 0.01~0.8 MPa	1
Air storage tank	C3513010-S0300	Volume: 25 L	1
Electric proportional valve	ITV2050-212L-X410	Input voltage: 0–10 V	1
ABS solenoid valve	3550010-R9800	DC24 V	1
Brake chamber	C3519D010A	Inlet joint diameter: 12 mm	1
Pressure sensor	PSE540A-R06	Measuring pressure range: 0~1.0 MPa	1
Differential pressure sensor	MB510	Differential pressure range: 0~20 KPa	1
Switching mode power supply	MW D-60B	Output voltage: DC 24 V/5 V	1

**Table 4:** Dimensional parameters for laminar flow resistance tubes.

Laminar Flow Resistance Tube Composition	Number	Inner Diameter (mm)	Outer Diameter (mm)	Length (mm)
Capillaries	54	0.6	1	100
Air ducts	1	9	10	100

**Table 5:** Key parameters for the electrical proportional valve simulation.

Name	Size	Unit	Name	Size	Unit	Size
Effective area at the top of the valve stem	4.13 × 10^−4^	m^2^	Intake spool spring pre-compression	2.4 × 10^−4^	m	4.13 × 10^−4^
Effective area at the bottom of the valve stem	3.27 × 10^−4^	m^2^	Exhaust spool spring pre-compression	1.6 × 10^−4^	m	3.27 × 10^−4^
Stem assembly mass	0.08	kg	Equivalent spring stiffness	220,000	N/m	0.08
